# BCG directly induces cell cycle arrest in human transitional carcinoma cell lines as a consequence of integrin cross-linking

**DOI:** 10.1186/1471-2490-5-8

**Published:** 2005-05-12

**Authors:** Fanghong Chen, Guangjian Zhang, Yoshiki Iwamoto, William A See

**Affiliations:** 1 Department of Urology, Medical College of Wisconsin, Milwaukee, WI, USA; 2 Human and Molecular Genetics Center, Medical College of Wisconsin, Milwaukee, WI, USA; 3 Co-first authors

## Abstract

**Background:**

Current models of the mechanism by which intravesical BCG induces an anti-tumor effect in urothelial carcinoma propose a secondary cellular immune response as principally responsible. Our group has demonstrated that BCG mediated cross-linking of α51 integrin receptors present on the tumor surface elicits a complex biologic response involving AP1 and NF-κB signaling as well as the transactivation of immediate early genes. This study evaluated the direct biologic effect of cross-linking α5β1 integrin on cell cycle progression and apoptosis in two human urothelial carcinoma cell lines.

**Methods:**

Two independent assays (MTT and Colony forming ability) were employed to measure the effect of α5β1 cross-linking (antibody mediated or BCG) on cellular proliferation. Flow cytometry was employed to measure effect of BCG and α5β1 antibody mediated cross-linking on cell cycle progression. Apoptosis was measured using assays for both DNA laddering and Caspase 3 activation.

**Results:**

Results demonstrate that integrin cross-linking by BCG, or antibody mediated crosslinking of α5β1 resulted in a decrease in proliferating cell number. BCG treatment or α5β1 cross-linking increased the percentage of cells in G0/G1, in both 253J and T24 cell lines. Peptide mediated blockade of integrin binding site using RGDS reversed the effect BCG on both proliferation and cell cycle arrest. Apoptosis in response to BCG was not identified by either DNA laddering or Caspase 3 activation.

**Conclusion:**

These findings show that BCG exerts a direct cytostatic effect on human urothelial carcinoma cell lines. Cell cycle arrest at the G1/S interface is a mechanism by which BCG inhibits cellular proliferation. This effect is duplicated by antibody mediated cross-linking of α5β1 and likely occurs as a consequence of crosslink-initiated signal transduction to cell cycle regulatory genes.

## Background

Bacille Calmette Guerin (BCG) remains the most effective available treatment option for non-muscle invasive urothelial carcinoma. Its superiority, both in terms of preventing recurrence and progression, has been demonstrated in multiple studies.[1-3] The mechanism responsible for BCG's superior anti-tumor activity is felt to be principally a consequence of an immune mediated response.[4] Investigators have shown the importance of a cellular immune response in orthotopic animal models of urothelial malignancy. The "effector" cell population responsible for the anti-tumor activity is currently felt to be natural killer (NK) cells.[4]

While an extensive literature supports an immune mechanism as being responsible for a portion of BCG's anti-tumor activity, a direct effect of BCG has been demonstrated by other authors. Multiple studies have demonstrated an in vitro anti-proliferative effect of BCG against human urothelial carcinoma cell lines.[5,6] Other authors have demonstrated a direct effect of BCG on other important biologic end points such as invasion.[7] The precise mechanism responsible for this direct effect is ill defined.

Work by others together with recent studies by our group has demonstrated that BCG has a direct gene regulatory effect in urothelial carcinoma cell lines. We have shown that this response is mediated via signal transduction initiated as a consequence of BCG induced cross-linking of α5β1 integrins present on the surface membrane of urothelial carcinoma cells.[8] Activation of signaling through NF-κB and AP1 initiate the transactivation of immediate early genes including interleukin 6 (IL-6).[9] Given the prevalence of NF-κB and AP1 response elements in the promoters of genes, it is likely that multiple genes are activated as a consequence of BCG/α5β1 cross-linking.

Historically, studies assessing a direct effect of BCG on tumor cells were hampered by the need to add a bacterial preparation to the culture media. In this setting it is difficult to separate a true direct anti-tumor effect of BCG from culture artifact.[6] Changes in pH, byproducts of the BCG preparation, and/or bacterial toxins have the potential to influence experimental outcome and yet fail to represent a relevant in vivo mechanism. The current study employed a non-biologic model that reproduces BCG induced signaling to determine whether BCG exerts a direct anti-tumor effect. Our results show that BCG decreases cell proliferation as measured by two separate assays of cell viability. Cell cycle arrest at the G1/S interface, rather than apoptosis, appears to be the mechanism by which this response occurs. These results are reproduced by the non-biologic signaling model in which α5β1 integrin receptors are cross-linked via antibodies and blocked by peptide fragments that inhibit the ability of fibronectin (FN) to function as a bridge for BCG mediated cross linking of these receptors.

## Methods

### Cell lines

The human transitional carcinoma cell lines 253J and T24 were obtained from American Type Cell Culture (Rockville, MD). Cells were maintained at 37°C, 5% CO_2 _in RPMI 1640 (Gibco BRL, Grand Island, NY) supplemented with 10% fetal bovine serum (FBS), penicillin, and streptomycin (complete media).

### Bacillus Calmette-Guerin (BCG)

TICE BCG, living organisms of an attenuated, Bacillus of Calmette and Guerin strain of Mycobacterium bovis were used in the experiments (Organon Inc, West Orange, NJ). Freeze dried BCG was reconstituted in complete media at an estimated concentration of 2.5 × 10^7 ^viable organisms/ml. (dilution assumed average viability of 4 × 10^8 ^organisms per vial based upon manufacturer's specified range of 1 to 8 × 10^8 ^per vial)

### Antibody mediated cross-linking

Antibody mediated cross-linking of α5β1 integrin was carried out as previously described.[8] Briefly, the following mouse mAbs were used: anti-α5 and anti-β1 Abs were purchased from Santa Cruz Biotechnology (Santa Cruz, CA). Affinity-purified F(ab')_2 _goat anti-mouse (GAM) antibody was purchased form BioSource International. Cells were resuspended in 10% FBS RPMI-1640 and cultured at 37°C for 45 min after trypsinization, then resuspended in RPMI-1640 serum-free medium (4 × 10^7 ^cells/300 μl/tube) and incubated with anti-5 or anti-1 integrin mAb (5 μg/ml) for 30 min at 4°C and washed with cold phosphate buffered saline (PBS). To initiate receptor cross-linking, F(ab')_2 _goat anti-mouse F(ab')_2 _Ab (100 μg/ml) was added after warming the cells to 37 C in RPMI-1640 medium Cells were incubated at 37C. Cells were harvested by pelleting the cells for 5 min at 500 × g.

### Cell viability assay (MTT)

For the determination of the cell viability, we estimated cellular bioreduction of 3-(4,5-dimethylthiazol-2-yl)-2,5-diphenyl tetrazolium bromide (MTT) subsequent to cell exposure to either BCG or antibody mediated α5β1 cross-linking.[10] A 200 μl volume of an exponentially growing cell suspension (1 × 10^4 ^cells/ml) was seeded into a 96-well microtiter plate. Twenty-four hours later 20 μl of BCG at a ratio of 50:1 BCG to cells was added. After incubation for 1, 2, 3, or 6 days at 37°C, 20 μl of MTT solution (5 mg/ml in phosphate buffered saline, PBS) was added to each well and the plates were incubated for a further 2 hr at 37°C. 200 μl of lysis buffer (20% SDS in 50% DMF) was added to each well and cells were continuously incubated for a further 2 hr at 37°C. Optical density was measured at 570 nm. Each experiment was performed in 3 replicate wells for each treatment and carried out independently a minimum of 3 times.

### Colony forming assay

Colony outgrowth of human urothelial carcinoma cells following exposure to BCG or antibody mediated cross-linking of α5β1 was measured using a colony forming assay. 1 × 10^5 ^of 253J or T24 cells were seed in 48-well plate with 1640 medium plus 10% FBS. After 24 hours cells were treated with 1 × 10^7 ^BCG. After 1, 2, 3, or 6 days incubation, the cells were trypsinized with 0.1 ml of Trypsin-EDTA per well. Trypsinized cells were resuspended in a 10 ml total volume of minimal essential medium (MEM) plus 20%FBS. 0.2 ml of this cell suspension was transferred into MEM plus 5%FBS at total volume of 25 ml. Five ml of this final cell suspension was seeded onto grid marked 60 mm culture plate with grid. Three plates were included for each group. The cells were incubated at 37°C. After 7 days of incubation, 0.5 ml of 2.5% glutaraldehyde (Sigma, St. Louis, MO) was directly added to each plate. The plates were incubated at room temperature for 45 min. The media and glutaraldehyde was aspirated and 1 ml of Wright-Giesma solution (EM Science, Gibbstown, NJ) added for 30 minutes to stain the colonies. One ml of Buffer Solution (LabChem Inc, Pittsburgh, PA) was added and the plates incubated at room temperature for an additional 30 min. The plates were gently rinsed under flowing tap water. After drying, the colonies were manually counted under magnification. For assay purposes, colonies were defined as consisting of 20 or more cells. For experiments employing antibody mediated cross-linking of α5 integrin, 1 × 10^5 ^of 253J or T24 cells were cultured in 48-well plate with 1640 plus 10% FBS at 37°C. After 24 hours, the media was changed to medium containing 5 μg/ml mouse monoclonal α5 antibody. The cells were incubated at 4°C for 45 min and then washed twice with serum free 1640 medium. Cross linking was achieved by incubating the cells with media containing GAM (10 μg/ml) at 37°C for 48 hrs. The CFA was performed as described as the above. Untreated cells, cells exposed to only primary antibody, and cells exposed to secondary antibody served as controls.

### Flow cytometry

Flow cytometry was employed to quantify the cell cycle compartmentalization of TCC lines following exposure to BCG or antibody mediated cross-linking of α5β1. 5 × 10^6 ^cells/per 6 cm dish were synchronized using serum starvation for 60 hours prior to the start of the experiment. At time zero, cells were exposed to BCG and 10% fetal bovine serum (FBS) at a ratio of 50:1 BCG to cells or to 10% FBS alone. After 24 or 48 hours cells were trypsinized, centrifuged at 1500 rpm for 5 min, washed with PBS, and then treated with 50 μg/ml RNase A (Sigma). DNA was stained with 50 μg/ml propidium iodide for 10 min at room temperature and cell cycle analysis performed.

### Apoptosis assay

Induction of apoptosis in response to BCG exposure or antibody mediated cross-linking of α5β1 was determined by measuring DNA fragmentation and activation of the Caspase 3 pathway.[11] For DNA fragmentation, 1 × 10^6 ^253J cells in 1640 plus 10%FBS were seeded in 6 cm plates. The following day cells were treated with 1 × 10^8^of BCG for 48 hrs. Non-adherent BCG was washed from the plate and cells harvested in a digestion solution, consisting of 100 mM NaCL, 10 mM Tris,10 mM EDTA, 0.5% SDS, and 0.1 mg/ml Proteinase K, pH8.0. After overnight digestion at 50°C, the cell lysates were extracted with phenol/chloroform and the DNA in the aqueous phase precipitated with ethanol and resuspended in Tris-EDTA buffer. After digestion with 5 μg/ml RNase A for 1 hr, DNA was re-precipitated and DNA samples were electrophoretically separated on 1.5% agarose gel containing ethidium bromide (0.5 μg/ml). DNA was visualized by a UV transilluminator and the gels photographed.

In the caspase 3 assay, 253J cells were seeded at 1 × 10^6 ^in 6 cm plates. The next day cells were treated with BCG at a ratio of 1 to 50 for 6 hours. At intervals following BCG exposure, cells were harvested with trypsinization, washed once in PBS, fixed and permeabilized using the Cytofix/Cytoperm Kit (BD Biosciences) for 20 min at room temperature (RT), pelleted and washed with Perm/Wash buffer. Cells were then stained with anti-active caspase-3 mAb using 20 ug/1 × 10^6 ^cells for 60 min at room temperature in the dark. Following incubation with the Ab, cells were washed in Perm/Wash Buffer and analyzed by flow cytometry.

All experiments were performed in triplicate. Results are shown as the average value +/- one standard error.

## Results

### BCG inhibits urothelial carcinoma cell proliferation

**Figure 1:**
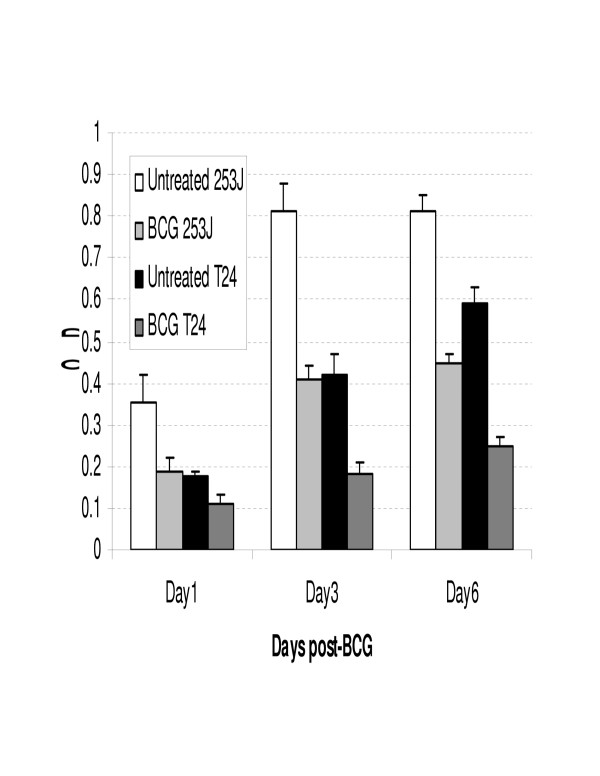
BCG decreases the proliferation of human urothelial carcinoma cell lines as measured by the MTT assay. Exposure of either 253J or T24 cells to BCG resulted in a decrease in viable cell number as measured by the optical density of the metabolic product. The day represents the duration of exposure to BCG

**Figure 2:**
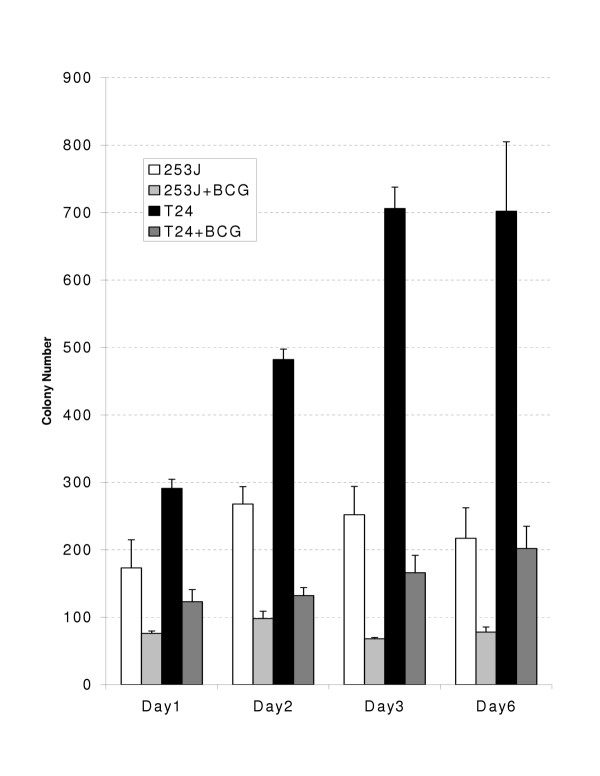
BCG decreases the proliferation of human urothelial carcinoma cell lines as measured by Colony Forming assay. Exposure of either 253J or T24 cells to BCG results in a time dependent decrease in viable cell number as measured by colony outgrowth. The day represents the duration of exposure to BCG.

Figures 1 and 2 demonstrate the effect of BCG exposure on the viability of the human urothelial carcinoma cell lines 253J and T24 as measured by the MTT and CF assays respectively. Both cell lines, in both assays, demonstrated a decrease in cell viability. Three days following BCG exposure, the viability of 253J and T24 cells was 41% and 58% of control values in the MTT assay. In the CF assay, colony counts were decreased in the 253J and T24 cells to 27% and 24 % respectively of control values after 3 days of exposure.

### Antibody mediated cross-linking of α5β1 integrin inhibits urothelial carcinoma cell proliferation

**Figure 3:**
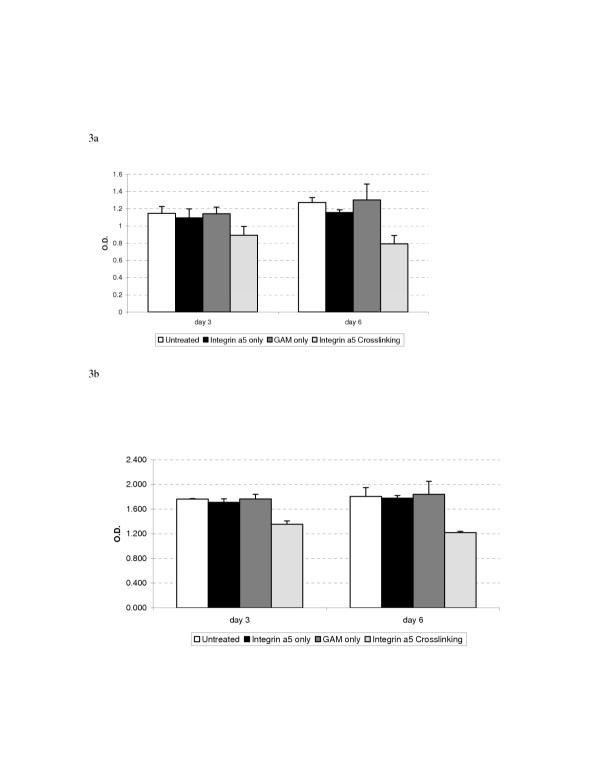
Antibody mediated cross-linking of a5b1 integrin decreases the proliferation of human urothelial carcinoma cell lines as measured by the MTT assay. Results are shown for a5 cross-linking in both 253J cells (figure 3a) and T24 cells (Figure 3b). Identical results were obtained when cross-linking was performed using antibodies to b1 (data not shown).

**Figure 4:**
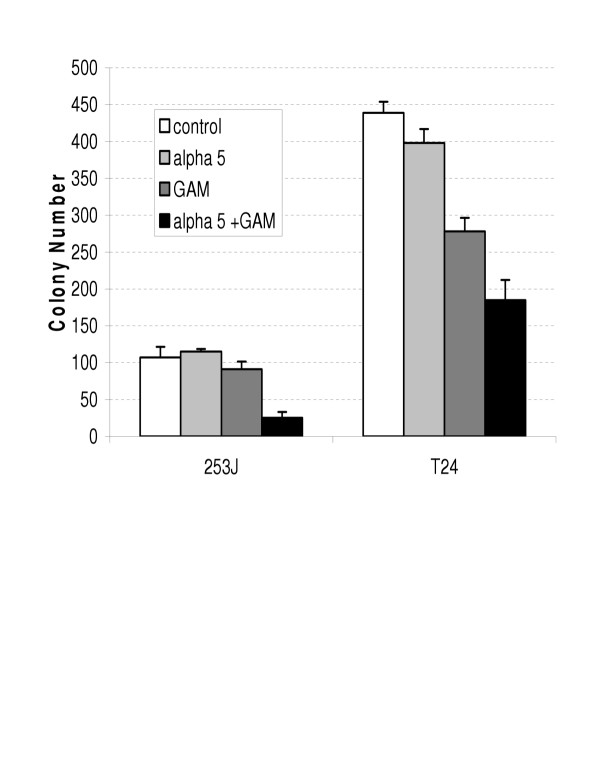
Antibody mediated cross-linking of α5β1 integrin decreases the proliferation of human urothelial carcinoma cell lines as measured by Colony Forming assay.

Figures 3 and 4 demonstrate the effect of antibody mediated α5β1 cross-linking on the viability of the human urothelial carcinoma cell lines 253J and T24 as measured by the MTT and CF assays respectively. Cross-linking decreased viability as measured by the MTT assay to 78% and 77% of untreated controls in the 253J and T24 lines at 3 days. Colony forming ability was similarly decreased in both cell lines with the average colony count reduced to 23% and 42% of untreated controls in the respective cell lines.

### BCG induces cell cycle arrest at the G1/S interface

**Figure 5:**
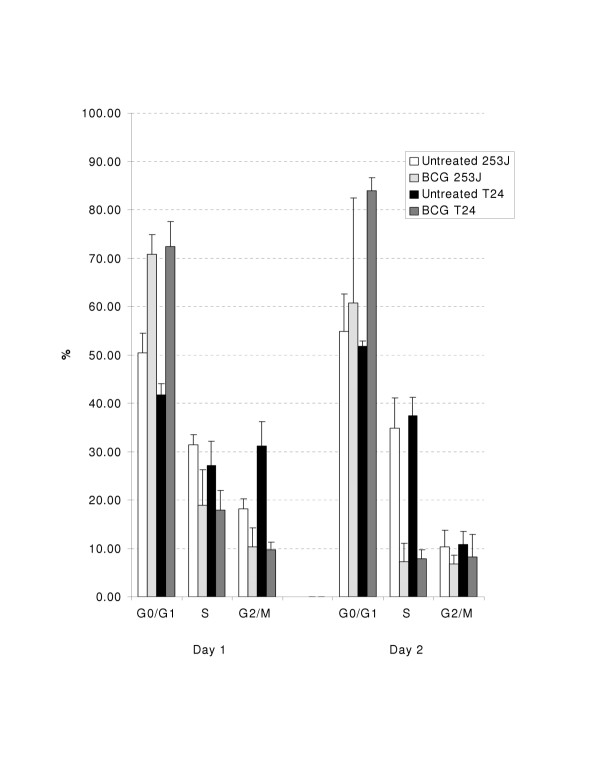
Human urothelial carcinoma cells exposed to BCG undergo cell cycle arrest at the G1/S interface. Both 253J and T24 cell lines increased the percentage of cells in G1, with a concomitant decrease in the S phase fraction, following BCG exposure.

Figure 5 demonstrates the effect of BCG exposure on cell cycle compartmentalization in 253J and T24 cells. Following BCG exposure, the percentage of cells in G0/G1 increased 40% and 75% in the respective cell lines. The increase in G phase population was mirrored by a decrease in the percent of cells in S phase. The S phase cell fraction in BCG treated cells represented 60% and 65% of the control groups in 253J and T24 cells respectively.

### Antibody mediated α5β1 integrin cross-linking induces cell cycle arrest at the G1/S interface

**Figure 6:**
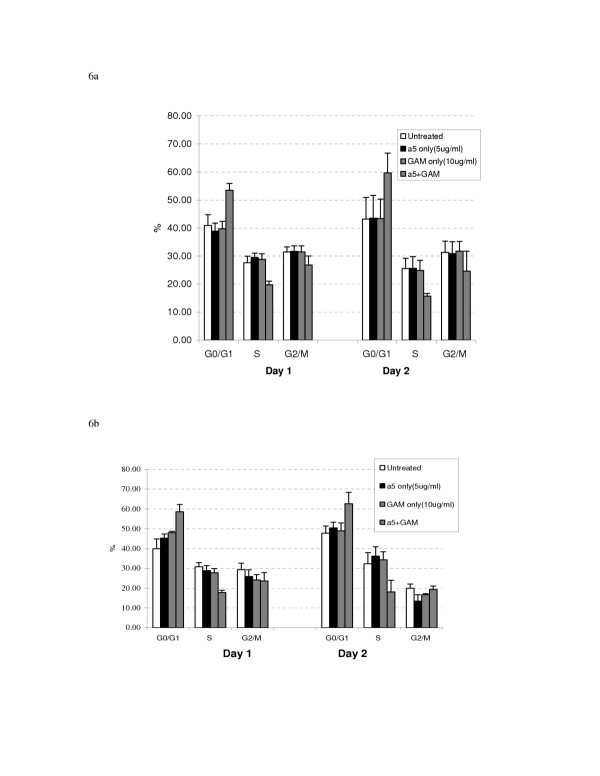
6a and 6b. Cell cycle compartmentalization in 253J (Figure 6a) and T24 (Figure 6b) cells following antibody mediated cross-linking of α5β1 integrin. Similar to the effect observed in response to BCG, antibody mediated cross-linking resulted in an accumulation of cells in G0-G1 with a concomitant decrease in the S-phase fraction.

Figure 6 demonstrates the effect of antibody mediated α5β1 cross-linking on cell cycle compartmentalization in 253J and T24 cells. Following cross-linking, the percentage of cells in G0/G1 increased 29% and 48% in the respective cell lines. The increase in G phase population was mirrored by a decrease in the percent of cells in S phase. The S phase cell fraction in cross-linked cells represented 71% and 60% of the control groups in 253J and T24 cells respectively.

### Peptide blockade of integrin fibronectin binding sites using RGDS reverses the effect of BCG on proliferation and cell cycle arrest

BCG attaches to the cell surface via a fibronectin bridge linking mycobacterial receptors to cell surface integrins.[12-14] Mycobacterial binding of multiple integrin bound fibronectin molecules can affect crosslinkage of integrin receptors.[8] Blocking the ability of FN to bind to either cellular or mycobacterial receptors precludes BCG attachment to the cell surface. [8,14] This series of experiments employed a competitive inhibitor of FN binding to integrins (RGDS) to assess the effect of inhibiting BCG binding to integrin receptors on the biologic response to BCG. While RGDS competitively inhibits the ability of FN to function as an opsonin for BCG binding (effectively inhibiting BCG/integrin interaction), prior studies have demonstrated that RGDS does not effect integrin mediated signaling initiated in response to antibody mediated cross linking of α5β1.[8] 253J cells were pre-incubated with RGDS or the control (non-blocking) peptide RGES for 1 hour prior to BCG exposure. The MTT and flow cytometric assays were then carried out as described above. Controls included each peptide alone, BCG alone, and untreated cells. In the MTT assay 2 concentrations of both the blocking and control peptides were employed (100 or 250 ug/ml). The 250 ug/ml concentration was used in the flow cytometry assay.

**Figure 7:**
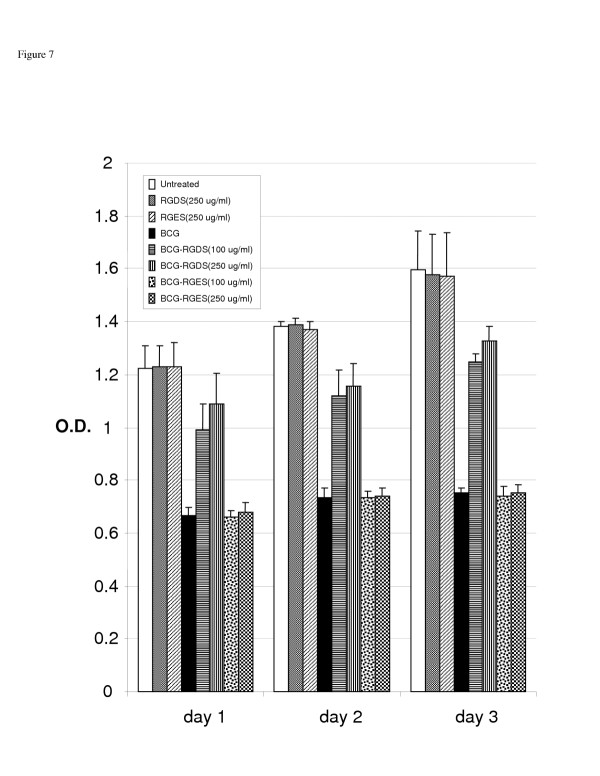
The effect of BCG on 253J Cellular Proliferation as measured by MTT assay. Peptide mediate integrin blockade (RGDS) reversed the anti-proliferative effect of BCG in a dose-response manner. The control peptide RGES had no effect.

**Figure 8:**
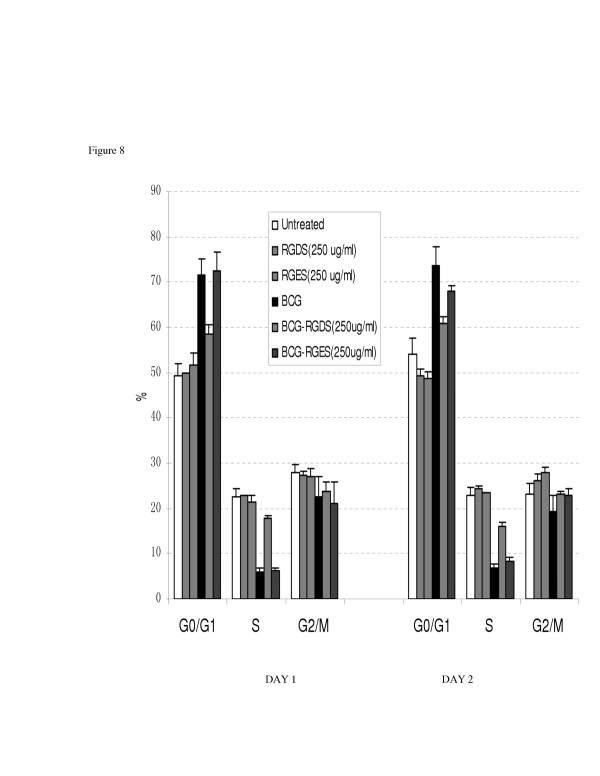
The effect of BCG on 253J cell cycle compartmentalization as measured by flow cytometry. Peptide mediate integrin blockade (RGDS) reversed the G1 cell cycle arrest effect of BCG. The control peptide RGES had no effect.

Figure 7 demonstrates the effect of integrin blockade on the anti-proliferative effect of BCG as measured by MTT assay. RGDS reduced the BCG effect in a "dose-response" manner with a maximal 4 fold inhibition at the 250 ug/ml concentration. RGES had no effect. In the flow cytometric assay, RGDS inhibited G1 arrest. The BCG induced increase in the G1 fraction was reduced by 60% in RGDS treated cells. RGES pretreatment had no effect on G1 or S phase fraction compared to controls (Figure 8).

### BCG does not induce an apoptotic response in urothelial carcinoma cell lines

A series of experiments were conducted to determine if BCG exposure induced apoptosis in either the 253J or T24 cell lines. Gel electrophoresis of cellular DNA obtained at intervals following BCG exposure failed to demonstrate the laddering associated with apoptosis. Flow cytometry for the apoptotic pathway enzyme caspase 3 failed to demonstrate activation of this enzyme in response to BCG. (Data not shown)

## Discussion

The anti-tumor effect of BCG is well established. Clinical experience, the results of animal models, as well as in vitro experiments provide data in clear support of this effect. While an anti-tumor effect of BCG is certain, the mechanism through which this occurs is less clear. In vivo animal models of BCG's anti-tumor effect suggest a critical role for a cell mediated immune response. At the same time, in vitro studies using human urothelial cell lines identify a direct effect of BCG. The relative contribution of these two possible mechanisms to BCG's clinical anti-tumor activity is unknown.

A number of studies suggest that a direct anti-tumor effect is at least in part responsible for BCG's activity. Work by Pryor, Ciao, and Sasaki all have reported a direct anti-proliferative effect of BCG.[5,6,15] Liu et al demonstrated that BCG, as a consequence of its interaction with cell surface bound fibronectin, abrogates invasion and motility of urothelial carcinoma cell lines.[7] Cellular internalization of BCG, with resultant alterations in reactive oxygen species and nitric oxide, has been proposed as a mechanism contributing to direct BCG mediated cytotoxicity. [16,17]

Fibronectin functions as an opsonin, linking BCG to cell surface fibronectin receptors of which α5β1 integrin is the predominate FN receptor on urothelial cells. This linkage is a requisite step for BCG's antitumor activity.[14] Rather than constituting a passive interaction, our prior reports have shown that FN mediated BCG adherence to the urothelial carcinoma surface has a pharmacogenetic effect as exemplified by transactivation of IL-6.[9] This effect is mediated through signal transduction pathways involving NF-κB and AP-1. BCG induced signaling and gene transactivation pathways are identical to those observed in response to antibody mediated cross linking of α5β1.[8] Strategies that prevent the ability of FN to function as an opsonin, including competitive inhibition of FN receptors using RGDS and simultaneous saturation of mycobacterial and cell surface FN receptors using excess exogenous FN, inhibit the signaling and transactivation responses to BCG.[8,18] The current report moves beyond the details of the signaling pathway to examine the biologic effect of BCG induced signaling on a tumor biologic end point with clear treatment relevance.

Consistent with what has been reported by others, our results demonstrate that BCG exerts a direct anti-proliferative effect on human urothelial carcinoma cell lines. The observed anti-proliferative effect does not appear to occur as a consequence of apoptosis. To our knowledge this report is the first to identify cell cycle arrest at the G1/S interface as a mechanism by which BCG exerts an anti-proliferative effect. As was the case for BCG induced signaling and gene transactivation, this effect was reproduced using antibody mediated cross-linking of α5β1 integrin. Elimination of the opsonin function of fibronectin using the peptide competitive inhibitor RGDS, effectively blocking the ability of BCG to bind integrin receptors, reversed the biologic effects of BCG. These findings, together with the results of prior studies, demonstrate that a portion of the biologic response to BCG occurs as a consequence of FN mediated binding of BCG to integrin receptors present on the urothelial cell surface. The failure of simple integrin ligation to simulate the BCG response, the ability of antibody mediated crosslinking of α5β1 integrin to duplicate the BCG response, the central role of FN in the biologic response to BCG, together with the predominance of α5β1 as the principal FN binding integrin on urothelial cells, strongly supports a model in which the biologic response of the tumor cell to BCG occurs as a consequence of BCG crosslinking of α5β1 integrin receptors.

The results of this study have direct clinical relevance. The demonstration that BCG has a direct anti-tumor effect opens this pathway for potential manipulation to improve treatment outcome. This is important given the fact that 30% of patients are BCG refractory, and that the long-term durability of response to BCG is limited. Finally, and equally important, the dissection of the pathway through which BCG exerts an anti-tumor effect will allow us to move further towards our ultimate goal of eliminating the need to expose patients to the risk of a viable biologic organism and perhaps allow the development of less toxic equally effective treatment approaches.

## Conclusion

BCG, as a consequence of integrin cross-linking, exerts direct anti-tumor effect against human urothelial carcinoma cell lines. Cell cycle arrest at the G1/S interface, rather than apoptosis, is a mechanism contributing to BCG's anti-proliferative effect. Additional studies will be required to define the molecular pathways that contribute to this response.

## Competing interests

The author(s) declare that they have no competing interests.

## Authors' contributions

FC carried out the flow cytometric experiments for cell cycle arrest and apoptosis and participated in drafting the manuscript. GZ performed the cell viability and colony forming assays and aided in drafting the manuscript. YI developed the protocols for flow cytometric experiments, aided in the design of the studies and drafted the manuscript. WAS conceived of the study, participated in its design and coordination, and drafted the manuscript. All authors read and approved the final manuscript.

## Pre-publication history

The pre-publication history for this paper can be accessed here:


